# Draft genome sequence data of maqui *(Aristotelia chilensis)* and identification of SSR markers

**DOI:** 10.1016/j.dib.2019.104545

**Published:** 2019-09-20

**Authors:** Adriana Bastías, Francisco Correa, Pamela Rojas, Constanza Martin, Jorge Pérez-Diaz, Cristian Yáñez, Mara Cuevas, Ricardo Verdugo, Boris Sagredo

**Affiliations:** a Facultad de Ciencias de La Salud, Instituto de Ciencias Biomédicas, Universidad Autónoma de Chile, Avenida Pedro de Valdivia 425, Providencia, Santiago, Chile; b Laboratorio de Biotecnología y Recursos Naturales, Instituto de Investigaciones Agropecuarias (INIA) CRI Rayentué, Av. Salamanca s/n, Sector Los Choapinos, Rengo, Chile; c Facultad Medicina Norte, Universidad de Chile, Av. Independencia 1027, Santiago, Chile

**Keywords:** Maqui, Aristotelia chilensis, Draft genome, Sequencing, Illumina NextSeq platform, SSR markers, Microsatellite

## Abstract

Maqui (*Aristotelia chilensis* [Molina] Stunz) is a small dioecious tree, belonging to the Elaeocarpaceae family. Maqui fruit has high levels of antioxidant activity, which are due to elevated anthocyanin and polyphenol content. Here we describe a draft genome sequence data of maqui (*A. chilensis*). The genomic sequence datasets were obtained using Illumina NextSeq platform. Nucleotide sequences of raw reads and the assembled draft genome are available at NCBI's Sequence Read Archive as BioProject PRJNA544858. Also, a total of 210067 microsatellite or simple sequence repeat (SSR) markers were identified.

**Table:** Specifications table

Subject area	*Genomics*
More specific subject area	*Plant Genomics*
Type of data	*Tables and figures*
How data was acquired	Paired-end–tag DNA sequencing was realized using illumina NexSeq 550 platform.
Data format	Raw and analyzed data of draft genome assembly; SSR table
Experimental factors	Leaves of maqui, DNA extraction and *do novo* sequencing.
Experimental features	Genomic DNA was extracted from leaves of maqui (*Aristotelia chilensis*) with the DNeasy Plant Mini Kit (QIAGEN, USA). The paired-end library was sequenced using Illumina NexSeq 550 plataform. *De novo* assembling was done with MaSuRCA software. SSR identification analysis was assessed with the MIcroSAtellite software.
Data source location	Rengo, Chile, INIA-Rayentue (Avda. Salamanca s/n, Km 105 ruta 5 sur, sector Los Choapinos). Latitude 34°19′16.1″S and longitude 70°50′03.6″W.
Data accessibility	The nucleotide sequences of raw reads and assembled draft genome are available at NCBI's Sequence Read Archive as BioProject PRJNA544858 (https://www.ncbi.nlm.nih.gov/bioproject/?term=PRJNA544858)
Related research article	Bastías, A., Correa, F., Rojas, P., Almada, R., Muñoz, C., Sagredo, B., 2016. Identification and Characterization of Microsatellite *Loci* in Maqui (*Aristotelia chilensis* [Molina] Stunz) Using Next-Generation Sequencing (NGS). PLoS ONE 11(7): e0159825. https://doi.org/10.1371/journal.pone.0159825

**Table:** 

**Value of the data** • Data of raw sequence reads and assembled draft genome of maqui (*Aristotelia chilensis*) contribute to establish a genomic platform for this plant species. • Draft genome data can facilitate the identification of molecular mechanisms that underlie properties of maqui products, thereafter contribute to improve them by classical and/or biotechnological approaches. • The draft genome data will accelerate functional genomics research in this species. • The newly developed SSR markers dataset of maqui should be useful tools to assesses its genetic diversity and understand its genetic structure, facilitating the implementation of effective conservation system of its natural populations.

## 1 Data

Here we described data of raw sequence-reads, an assembled draft genome and SSR analysis from genomic DNA of maquí (*A. chilensis*). Both raw data and assembled draft genome are available at NCBI's Sequence Read Archive as BioProject PRJNA544858P (https://www.ncbi.nlm.nih.gov/bioproject/?term=PRJNA544858). The genomic DNA was obtained from fresh leaves of maqui. Using a library with 300 bp insert size and paired-end–tag DNA sequencing using illumina NextSeq 550 platform around 187 million 2 × 151 bp reads were generated. After a process of quality trimming and filtering of data using FastQC v0.11.5, which allow to remove reads containing more than 5% unknown nucleotides, low-quality reads (reads containing more than 50% bases with Q-value ≤ 20), all unpaired reads and short reads (<35 bp), a 95.87% from the total reads were suitable for genome assembling (Table 1). A draft genome of maqui was obtained through *de novo* assembling using MaSuRCA software [1] (see Table 2).

**Table 1:** Dataset of maqui *(A. chilensis)* reads obtained by Illumina NextSeq 550 sequencing before and after filtering.

Species	Before filtering		After filtering		
	Total reads (×2)	GC (%)	Total reads (×2)	GC (%)	% total reads
*A. chilensis*	187,132,040	36	179,407,345	35.13	95,87

**Table 2:** Data on contig measurements that were assembled by MaSuRCA software with high-quality reads.

Item	Number	Description
Total number of sequences	58,451	Counts
N50	13,213	A + T + C + G + N (bp)
Max contig	113,184	(A + T + C + G) not include Ns
Min contig	500	(A + T + C + G) not include Ns
Total length of sequences	326,414,674	A + T + C + G + N (bp)
Total valid length of sequences	326,169,547	A + T + C + G (bp)
Unknown bases (Ns) in sequences	245,127	bp
Percentage of unknown bases	0.08	Percentage (%)
GC content	35.13	(G + C)/(A + T + C + G) not include Ns (%)

The final genome assembly had a total length of 326 Mb, comprising in 58,451 scaffolds and 140X of mean coverage were obtained. The scaffold N50s of this assembly were 13.2 kb, and unclosed gap regions represented 0.08% of the assembly. In addition, the G + C content of the genome assembly excluding gaps was estimated to be 35.13%. The assembled draft genome was constructed using 343,326,678 (95.68%) of the raw sequence reads.

To check the draft genome generated, the raw sequence reads for transcriptomic data from maqui were downloaded from NCBI database (BioProject PRJNA255387) and mapped to the draft genome using HiSAT2 map alignment program [2] with 93.61% of filtered RNA sequences were mapped.

**Fig. 1:**
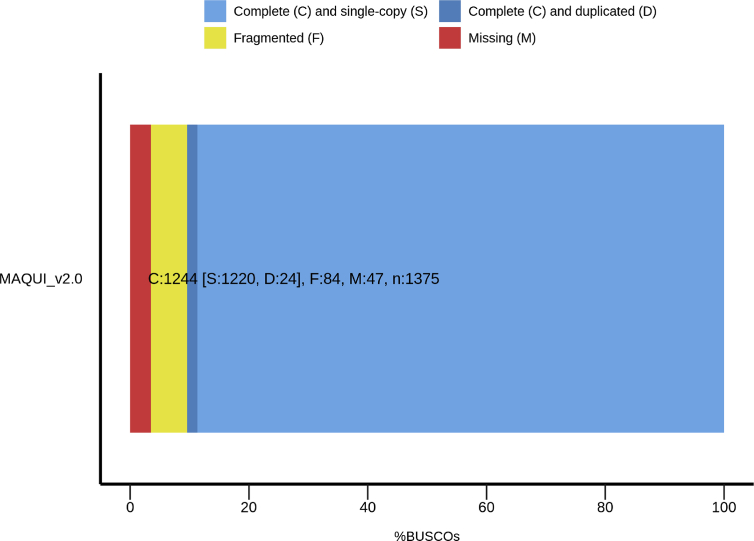
Percentage of 1375 single-copy orthologs genes from 60 plants by BUSCO analysis.

The assembled *A. chilensis* draft genome was analyzed with BUSCO tools [3] using the embryophyta database (Fig. 1). We found 1244 complete orthologs genes (C: 90.4%), 1220 orthologs complete genes and single-copy (S: 88.7%), 24 orthologs complete genes and duplicated (D: 1.7%), 84 orthologs fragmented genes (F: 6.1%) and 47 missing genes BUSCO's (M: 3.5%).

**Fig. 2:**
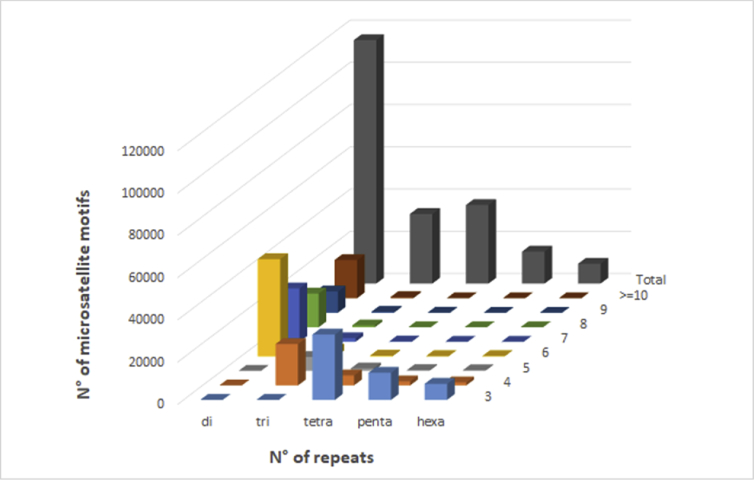
Distribution of SSR from maqui (*A. chilensis*) with Di-to Hexa-nucleotides by repeat numbers. The graph is based on a total of 210,067 SSRs detected in non-redundant genomic maqui DNA. Di, tri, tetra, penta and hexa, refer to dinucleotides, trinucleotides, tetranucleotides, pentanucleotides, and hexanucleotides, respectively.

The assembled draft genome of maqui was used to identify microsatellite sequences or simple sequence repeat (SSR) (Table 3). Dinucleotide to hexanucleotide repeat microsatellite sequences, with repeat motif size ranging from 2 to 6 bp and a length ≥12 bp were considered. This includes data of dinucleotide repeats ≥6, trinucleotide repeats ≥4, and tetra-, penta- and hexa-, repeats ≥3. A total of 210.067 maqui perfect SSR markers were identified (Table 3). Among the identified SSRs, dinucleotide motifs (54.87%) were the most common, followed by tetranucleotide (17.73%) and trinucleotide motifs (15.7%) (Table 4). We also examined the distribution of maqui microsatellites with regard to motif length and type and the number of repeats (Fig. 2). A total of 111,531 primer pairs were designed from flanking sequences of di-to hexanucleotide microsatellites of maqui (*A. chilensis*) and are available in Table S1.

**Table 3:** Dataset of microsatellite (SSRs) searches of *maqui (A. chilensis)* using PERF software.

Item	Number	Description
Total number of perfect SSRs	210,067	Counts
Total length of perfect SSRs	3,153,200	bp
The average length of SSRs	15.02	total ssr length/total ssr counts (bp)
SSRs per sequence	4	total SSR counts/sequence counts
% of sequence occupied by SSRs	0.97	ssr total length/total sequence size (%)
Relative abundance	644.04	total SSRs/total valid length (loci/Mb)
Relative density	9667.36	total SSR length/total valid length (bp/Mb)

**Table 4:** Distribution to microsatellites di-to hexanucleotide motifs in the assembled genomic DNA of maqui *(A. chilensis)*.

Type	Counts	Length (bp)	Percent (%)	Relative Abundance (loci/Mb)	Relative Density (bp/Mb)
Di	115,254	1,765,324	54.87	353.36	5412.29
Tri	32,972	480,600	15.7	101.09	1473.47
Tetra	37,247	481,296	17.73	114.2	1475.6
Penta	15,190	242,440	7.23	46.57	743.29
Hexa	9,404	183,540	4.48	28.83	562.71

## 2 Experimental design, materials, and methods

### 2.1 Plant material

Young maqui (*A. chilensis*) leaves were collected at INIA-Rayentue, Rengo, O'Higgins Region, Chile, (Latitude 34°19′16.1″S and longitude 70°50′03.6″W). Samples were frozen in liquid nitrogen and stored at −80 °C until DNA extraction and subsequent analysis.

### 2.2 Genomic DNA extraction

Genomic DNA of maqui (*A. chilensis*) was extracted as was described by Bastias et al., 2016 [4] using DNeasy Plant Mini Kit (Qiagen) following the manufacturer's instructions.

### 2.3 DNA sequencing

Paired-end–tag DNA *de novo* sequencing using Illumina NextSeq 550 platform was used. Approximately 187 million 2 × 151 bp reads were generated from library with 300 bp insert size. Sequence quality of raw genomic data was assessed using FastQC v0.11.5 software (http://www.bioinformatics.babraham.ac.uk/projects/fastqc). Quality trimming and filtering of data was performed using fastqp (https://github.com/OpenGene/fastp) [5], reads containing more than 5% unknown nucleotides, and low-quality reads (reads containing more than 50% bases with Q-value ≤ 20) and all unpaired reads were discarded. Short reads (<35 bp) were removed from the filtered data.

### 2.4 Genome assembly

Then *de novo* assembly of the clean reads was performed to generate contigs and scaffolds. For *de novo* assembly we used MaSuRCA (http://www.genome.umd.edu/masurca.html) [1] with optimized k-mer length of 85, calculated by KmerGenie software [6]. Assembly statistics were obtained with QUAST (quality assessment tool for genome assemblies) software [7].

### 2.5 Assessing genome assembly completeness with benchmarking universal single-copy orthologs (BUSCO)

The assembled *A. chilensis* genome data was searched for BUSCO analysis [3] against the embryophyta database, consisting of 1375 orthologs constructed from 60 species.

### 2.6 Identification of Putative SSRs and primer design

We analyzed perfect SSRs. The contig sequences obtained in FASTA files were screened with a repeat motif size range of 2–6 bp and a length of >12 bp. This includes dinucleotide repeats ≥6, trinucleotide repeats ≥4, and tetra-, penta- and hexa repeats ≥3, using PERF software [8]. The program allows for direct primer design using PRIMER 3 [9] by searching for microsatellite repeats and primer annealing sites in the flanking regions.
